# Methods for Characterizing Intercalation in Aqueous Zinc Ion Battery Cathodes: A Review

**DOI:** 10.1002/advs.202303211

**Published:** 2023-07-09

**Authors:** Ian Rongde Tay, Junmin Xue, Wee Siang Vincent Lee

**Affiliations:** ^1^ Department of Materials Science and Engineering National University of Singapore. Block E3A #03‐14 7 Engineering Drive 1 Singapore 117574 Singapore

**Keywords:** ab initio methods, aqueous zinc ion batteries, battery cathodes, characterization methods, experimental methods, intercalation

## Abstract

Aqueous zinc ion batteries have gained research attention as a safer, economical and more environmentally friendly alternative to lithium‐ion batteries. Similar to lithium batteries, intercalation processes play an important role in the charge storage behaviour of aqueous zinc ion batteries, with the pre‐intercalation of guest species in the cathode being also employed as a strategy to improve battery performance. In view of this, proving hypothesized mechanisms of intercalation, as well as rigorously characterizing intercalation processes in aqueous zinc ion batteries is crucial to achieve advances in battery performance. This review aims to evaluate the range of techniques commonly used to characterize intercalation in aqueous zinc ion battery cathodes, providing a perspective on the approaches that can be utilized to rigorously understand such intercalation processes.

## Graphical abstract

Aqueous zinc ion batteries have gained research attention as safer, more economical and more environmentally friendly alternatives to lithium‐ion batteries. This review aims to evaluate the range of techniques applied to characterize intercalation in aqueous zinc ion battery cathodes, providing a perspective on the approaches that can be utilized to rigorously understand such intercalation processes essential to battery performance.

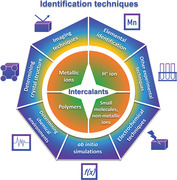

## 1 Introduction

In order to address the safety and environmental issues surrounding the ubiquitous use of lithium‐ion batteries today, aqueous zinc ion batteries as a safer, economical, and more environmentally friendly^[^ ^1^ ^]^ alternative has gained growing research attention in the last decade.^[^ ^1^, ^2^ ^]^ To improve the performance of zinc ion batteries, many groups have worked on the synthesis of high‐performance cathode materials, with vanadium‐based materials^[^ ^2^, ^3^ ^]^ and manganese‐based materials^[^ ^3^, ^4^ ^]^ exhibiting charge storage capabilities. In aqueous zinc ion batteries, intercalation is one of the key mechanisms for charge storage, most commonly involving the intercalation of Zn^2+^ ions,^[^ ^3^, ^4^, ^5^ ^]^ and often H^+^ ions^[^ ^3^, ^4^, ^5^ ^]^ into the cathode during discharge and removal of these species during charging. Due to the important role played by intercalation in zinc ion battery operation, it would be prudent to be thorough in investigating intercalation processes that occur at the cathodes of zinc ion batteries. Understanding the mechanism and kinetics of Zn^2+^ intercalation into cathode materials would also prove useful in the design of high‐performance cathode materials. Present challenges such as cathode dissolution leading to poor cyclic stability^[^ ^1^, ^3^, ^4^, ^5^ ^]^ especially for materials such as MnO_2_,^[^ ^2^, ^4^ ^]^ as well as poor reaction kinetics of Mn‐based^[^ ^4a^ ^]^ and V‐based^[^ ^3a^ ^]^ materials leading to low energy density at high current densities are attributed to the strong interactions formed between cathode materials and the Zn^2+^ ions intercalated into the cathode.^[^ ^1^, ^3^, ^5^, ^6^ ^]^ Hence, insight into the behaviour of Zn^2+^ during intercalation could lead to innovations that reduce the energy barrier and improve the reversibility of zinc ion intercalation, leading to improved cathode performance. To achieve this, many research groups have synthesized cathode materials that contain pre‐intercalated guest species,^[^ ^4^, ^5^ ^]^ introduced in order to improve kinetics and cyclic performance, hence understanding intercalation is made even more crucial and important. For research groups focusing on designing cathode materials for zinc ion batteries, observing definite proof of intercalation during battery performance and during any pre‐intercalation is therefore indispensable in accounting for the experimental behaviour of the cathode.

**Figure 1:**
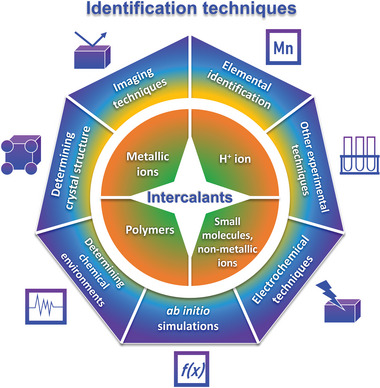
Overview of common intercalants and identification techniques in aqueous zinc ion battery cathodes.

However, no single experimental technique available is able to provide a full picture of intercalation. Techniques such as X‐ray diffraction can reveal changes in material structure, but cannot directly identify the presence of the intercalant, while elemental identification methods such as energy dispersive X‐ray spectroscopy can provide proof of the presence of the intercalant, but not its distribution at greater depths below the surface. Hence, current works often utilize a combination of techniques to prove successful intercalation, combining visual evidence, detection of changes in the material crystal structure, detection of changes in the chemistry of the material such as average oxidation states and spectroscopic behaviour, and computational simulations. Despite increasing research attention on zinc ion batteries, there has been no present work reviewing the success of experimental approaches to prove intercalation in cathode materials. We believe that research groups focusing on zinc ion batteries will benefit from a guideline that suggests approaches for obtaining concrete evidence of intercalation. Herein, this review will evaluate the suitability of various techniques in identifying intercalation and aim to answer the question of what combinatory approaches could provide convincing evidence of intercalation. We will first discuss the proposed mechanisms and chemistry of intercalation in zinc ion batteries, briefly covering intercalation into the cathode as both a charge storage mechanism as well as a strategy for the enhancement of cathode performance. In the following section, we will look into the techniques that have been utilized to investigate intercalation in zinc ion battery cathodes, with 7 main categories of methods categorized based on their working principles (**Figure** **1**). Subsequently, we will discuss the techniques utilized to identify the intercalation of different types of intercalants, which fall into 4 broad categories (Figure 1). Last, we will provide our perspective and outlook on this crucial aspect of zinc ion battery research.

## 2 Intercalation in Zinc‐Ion Battery Cathodes

Intercalation of species into the cathode material can be divided into two general categories: 1) Intercalation of Zn^2+^/H^+^ during the discharge process to achieve charge storage behaviour for battery performance and 2) intentional pre‐intercalation of guest species into the cathode to enhance battery performance. During the discharge of aqueous zinc ion batteries, Zn^2+^ intercalation into the cathode is known to contribute to charge storage,^[^ ^3^, ^4^, ^5^ ^]^ in which Zn^2+^ inserts into the lattice spacing of the cathode. In some cathode materials, especially oxides,^[^ ^5a^ ^]^ H^+^ intercalation can also occur during discharge, causing changes in electrolyte pH and the resultant formation of by‐products.^[^ ^3^, ^4^, ^5^ ^]^

**Figure 2:**
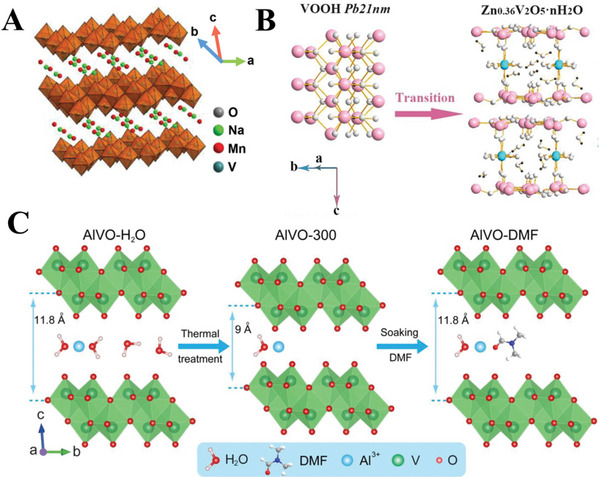
Crystal structures of selected examples of zinc ion battery cathodes. A) Structure of the sodium vanadium oxide cathode intercalated with Mn. Reproduced with permission.^[^ ^13^ ^]^ Copyright 2020, Wiley‐VCH. B) Schematic of the vanadium oxide cathode material before and after the intercalation of Zn^2+^. Reproduced with permission.^[^ ^9^ ^]^ Copyright 2022, Elsevier. C) Schematic of the structures of the aluminium vanadium oxide cathode material intercalated with water and with *N, N*‐dimethylformamide (DMF). Reproduced with permission.^[^ ^14^ ^]^ Copyright 2021, Wiley‐VCH.

The intercalation of Zn^2+^ can result in an expansion in the interlayer spacing of the cathode^[^ ^7^ ^]^ due to the increased size of the Zn^2+^ cation caused by the solvation shell of water molecules surrounding it (≈2.1 Å, compared to 0.88 Å for unsolvated Zn^2+^ with a coordination number of 6^[^ ^8^ ^]^). In other materials, a detectable contraction^[^ ^9^, ^10^ ^]^ (≤0.5 Å^[^ ^12b,e^ ^]^) in the lattice spacing of the cathode material is instead observed, owing to interactions formed between the bivalent Zn^2+^ cation and electron‐rich centers in the material.^[^ ^12b–e^ ^]^ The changes in the interlayer spacing caused by the intercalation of Zn^2+^ affect cathode stability and ion (de)intercalation, thus influencing battery performance. For instance, Zhang et al. report that the intercalation of Zn^2+^ into a vanadium oxide cathode formed a stable, low crystallinity structure which reduced kinetic barriers for further zinc ion intercalation, improving the charge storage capacity in subsequent cycles (**Figure** **2**B).^[^ ^9^ ^]^ As such, it has been acknowledged that energy barriers for zinc ion diffusion in the cathode,^[^ ^7^, ^10^, ^11^ ^]^ and also the availability of storage sites in the cathode for the insertion of zinc,^[^ ^5^, ^10^, ^12^ ^]^ contribute to the kinetics and reversibility of zinc ion intercalation during discharge, thus influencing battery performance.

Therefore, reducing the energy barriers for zinc ion diffusion and increasing the area of storage sites in the cathode is a possible means to improve battery performance, and achieving this via pre‐intercalation of guest species into the cathode has been explored.^[^ ^4^, ^5^ ^]^ This pre‐intercalation of guest species such as alkali metals^[^ ^7c^ ^]^ and small molecules^[^ ^10a^ ^]^ modulates the interlayer spacing of the cathode and stabilizes the cathode material over numerous charge–discharge cycles.^[^ ^5f^ ^]^ For example, Du et al. report that the intercalation of metallic ions such as Mn, Fe, Co, Ni, Ca and K into a V_8_O_20_ cathode improved capacity relative to a NaV_3_O_8_ cathode, with the intercalation of Mn^2+^ improving capacity retention over 1000 cycles.^[^ ^13^ ^]^ The pre‐intercalation of molecules like ammonia and organic nitrogen compounds has also been explored to improve cathode performance^[^ ^10^, ^11^, ^14^, ^15^ ^]^ with improved cycling stability and energy storage capacity.

As intercalation is a process of crucial importance in zinc ion batteries, the identification and characterization of intercalants is key to understand battery behaviour. In the following section, we will discuss the characterization techniques that have been used to identify and quantify the presence of intercalants in zinc ion battery cathodes.

## 3 Overview of Characterization Methods for Intercalants

Methods to identify the presence of intercalants include elemental identification techniques such as energy dispersive X‐ray spectroscopy (EDS) and inductively coupled plasma atomic emission spectroscopy (ICP‐AES). In addition to identifying the presence of the intercalant, further methods are often employed to understand the chemical environment of the intercalants via methods like X‐ray photoelectron spectroscopy (XPS), as well as any changes in crystal structure upon intercalation using methods such as X‐ray diffraction (XRD). Electrochemical methods can further reveal how intercalation influences electrochemical properties, which is of importance for battery performance. Computations using ab initio density functional theory (DFT) models can also shed light on the theoretical mechanisms that influence ion diffusion and intercalation.

Characterization methods for intercalants can be broadly categorized as 1) imaging techniques, 2) elemental identification techniques, 3) techniques to determine crystal structures, 4) techniques to determine chemical environments, 5) other experimental techniques, 6) electrochemical techniques, and 7) ab initio simulations.

### 3.1 Imaging Techniques

**Figure 3:**
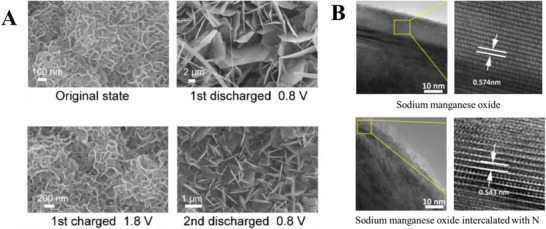
Examples of the utilization of SEM and HRTEM to identify intercalation. A) SEM images of MnO_2_ cathode pre‐intercalated with sulfur after discharge. Reproduced with permission.^[^ ^11g^ ^]^ Copyright 2022, Elsevier. B) HRTEM of sodium manganese oxide and nitrogen‐doped sodium manganese oxide. Reproduced with permission.^[^ ^20^ ^]^ Copyright 2022, American Chemical Society.

Zhao et al. observed under SEM the formation of nanoflakes of zinc hydroxide salt on their sulfur‐doped MnO_2_ cathode during discharge, which was morphologically different from the nanoflower structure of the cathode before discharge (**Figure** **3**).^[^ ^11g^ ^]^ Similarly, Bi et al. developed a sodium vanadate cathode pre‐intercalated with PEDOT and observed large flakes on the surface of the discharged electrode, which was confirmed with EDS and XRD to be a phase containing zinc hydroxide.^[^ ^16^ ^]^ Additionally, Liu et al. utilized SEM and EDS elemental mapping to identify the presence of a zinc hydroxide triflate salt on the surface of their nickel vanadium oxide cathode after discharge.^[^ ^10e^ ^]^ Based on the previous works,^[^ ^17^ ^]^ the formation of zinc hydroxide compounds was used as an indication of an increase in electrolyte pH, which in turn arises due to H^+^ intercalation into the cathode, hence the identification of zinc hydroxide salts through SEM and EDS has been used to provide indirect evidence that H^+^ intercalation had occurred. Generally, it appears that the use of SEM, combined with EDS, can identify morphological and elemental changes on the cathode surface, which when combined with the work of previous research, can act as indirect evidence of ion intercalation. SEM is a microscopy technique which involves the focusing of an electron beam on a material, with the image being formed from the signals of secondary electrons ejected by the material, or backscattered electrons reflected back from the material. SEM allows for the imaging of any morphological changes in the cathode during intercalation, or the formation of solid‐electrolyte interfaces and other by‐products on the cathode surface, and provides a high resolution of <1 nm and allows for the combination of elemental mapping instruments such as EDS.^[^ ^18^ ^]^ However, the penetration depth of the SEM electron beam is in the range of hundreds of nanometres to a few micrometers,^[^ ^19^ ^]^ restricting characterization to the surface of the material. This would make it challenging to utilize SEM to demonstrate intercalation in the bulk material at greater depths.

In addition to SEM, Li et al. utilized high‐resolution transmission electron microscopy (HRTEM) to quantify an increase in interlayer spacing from 0.62 to 0.96 nm after N‐doped carbon species was intercalated into their MoS_2_ cathode.^[^ ^11b^ ^]^ In another work, Cheng et al. utilized HRTEM to identify a change in the interlayer spacing of their sodium manganese oxide cathode from 0.574 to 0.543 nm upon the intercalation of nitrogen‐containing species (Figure 3).^[^ ^20^ ^]^ Similar to these works, many other studies have also used HRTEM to investigate the interlayer spacing of a layered material,^[^ ^10^, ^11^, ^15^, ^16^, ^21^ ^]^ which often changes upon intercalation, thus acting as indirect evidence for intercalation. HRTEM also allows for directly identifying intercalant atoms in the interlayer spacing, with Chen et al. utilizing HRTEM to identify the presence of intercalated Mn in their vanadium oxide cathode.^[^ ^11a^ ^]^ Transmission electron microscopy techniques involve the penetration of an electron beam through a thin sample, with the transmitted electrons being used to construct an image of the sample which can reach resolutions of a single layer of atoms, with HRTEM allowing for the imaging of single atoms and visual identification of crystal defects. Liu et al. utilized scanning transmission electron microscopy (STEM) to image their vanadium oxide cathode intercalated with Ni, identifying and quantifying the Ni–V internuclear distance,^[^ ^10e^ ^]^ with multiple works utilizing STEM in conjunction with EDS to obtain a map of the elemental composition of zinc ion battery cathodes.^[^ ^7^, ^10^, ^11^, ^21^ ^]^ An STEM operates similarly to a conventional TEM, but instead of capturing the image using an electron beam focused on a single spot, it scans the electron beam across the sample to construct the image.

### 3.2 Elemental Identification Techniques

**Figure 4:**
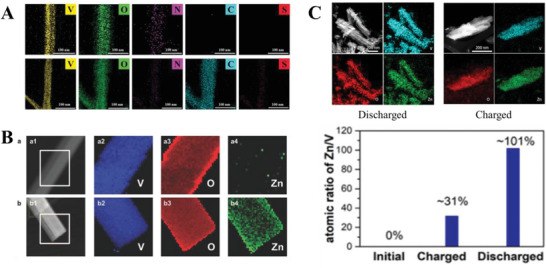
Examples of elemental identification techniques used to identify intercalation. A) EDS mapping of ammonium vanadate unmodified (top) and intercalated with PEDOT (bottom). Reproduced with permission.^[^ ^10c^ ^]^ Copyright 2021, Wiley‐VCH. B) EELS elemental mapping of H_2_V_3_O_8_ cathode in pristine condition and after discharge to 0.2 V. Reproduced with permission from.^[^ ^23^ ^]^ Copyright 2018, Wiley‐VCH. C) EDS mapping of the potassium vanadate cathode in the discharged and charged states, and atomic ratios of Zn: V in the various states. Reproduced with permission.^[^ ^7c^ ^]^ Copyright 2021, American Chemical Society.

Elemental identification techniques can include 1) identifying elements on the surface of the material or within the interlayer spacing, and 2) quantifying elemental composition of just the surface layer or the bulk material. Zong et al. utilized EDS to identify K and N elements in their potassium ammonium vanadate cathode after discharge and charging, indicating that for their cathode material, pre‐intercalated potassium and ammonium ions remain in the interlayer spacing during battery performance.^[^ ^10b^ ^]^ Kim et al. used EDS to confirm the presence of carbon in their ammonium vanadate cathode after the intercalation of PEDOT (**Figure** **4**).^[^ ^10c^ ^]^ Similar to Zong et al. and Kim et al., EDS has often been used to identify elemental changes on the material surface as direct evidence of the intercalation of Zn^2+^ during discharge or the pre‐intercalation of metal ions into the cathode,^[^ ^7^, ^9^, ^10^, ^11^, ^12^, ^21^, ^22^ ^]^ and indirect evidence of H^+^ intercalation through the identification of zinc hydroxide salts.^[^ ^10^, ^11^, ^16^, ^21^ ^]^ EDS utilizes an electron beam to produce X‐rays with energies characteristic of the elements present, and due to the small penetration depth of the electron beam as previously mentioned, EDS can only identify elements on the surface of the material. The elemental composition can be quantified with EDS based on the intensities of the peaks corresponding to each element. As aforementioned, EDS is often combined with microscopic techniques such as SEM to produce a visual map of the elements present on the material surface.

Instead of utilizing SEM‐EDS, Pang et al. conducted elemental mapping on their H_2_V_3_O_8_ nanowires cathode using electron energy loss spectroscopy (EELS) (Figure 4), observing no Zn signal in the pristine cathode, but the appearance of Zn during discharge. Additionally, they found a greater abundance of Zn intercalation on the surface and along the edge of their nanowires.^[^ ^23^ ^]^ While EDS has often been used with SEM, EELS is a technique that has been often combined with STEM imaging.^[^ ^24^ ^]^ In EELS, the electron beam is passed through the sample, and the energy loss of the electrons is measured. The energy loss corresponds to the interactions between the electron and the material, such as the excitation of phonons and the ionization of inner shell electrons. Based on the energy loss spectrum, the composition and structure of the sample can be deduced. Kim et al. used EELS to characterize their cellulose/carbon‐MnO_2_ cathode after Zn^2+^ intercalation and deintercalation during cycling and observed a shift in the EELS spectrum corresponding to a decrease in the average oxidation state of Mn, indirect evidence for the formation of MnO by‐product which was further confirmed with time‐of‐flight secondary ion mass spectrometry analysis.^[^ ^25^ ^]^ This could be achieved as EELS spectra not only allow for elemental identification but also characterization of the bonding and chemistry of the material, such as oxidation states and coordination environments.^[^ ^24^ ^]^

Qiu et al. investigated the ratio of K to V in their potassium vanadate cathode during cycling, supplementing their EDS results (Figure 4) with ICP‐AES results which found a K: V ratio of 0.2293 for the pristine cathode and 0.0022 for the cycled cathode, suggesting intercalation of Zn^2+^, and substitution of K^+^ with Zn^2+^.^[^ ^7c^ ^]^ Wan et al. also utilized ICP‐AES to quantify the aluminium content in a vanadium oxide cathode pre‐intercalated with Al^3+^,^[^ ^14^ ^]^ and a recent work by Yang et al. utilized ICP‐AES to determine the K: Mn ratio in their potassium intercalated manganese oxide cathode.^[^ ^26^ ^]^ Many other studies have utilized ICP‐AES to identify and quantify the intercalated element in aqueous zinc ion battery cathode materials.^[^ ^10^, ^11^, ^12^, ^21^, ^27^ ^]^ In contrast to surface techniques, ICP‐AES is a bulk technique that involves the use of inductively coupled plasma to excite atoms and ions in the sample, with the resultant emission of electromagnetic radiation at characteristic wavelengths allowing for the identification of elements present in the sample. In the process of ICP‐AES, the bulk sample is excited and its emission spectrum analyzed, hence information such as uneven elemental distribution on the surface, or differing elemental composition on the surface versus the bulk material are consequently lost. Users should therefore exercise caution in generalizing ICP‐AES results, as the presence of the intercalated element in the ICP‐AES result may not indicate intercalation into the bulk material, as it may only be present on the material surface. Likewise, ICP‐AES can only provide the average stoichiometric ratio of the elements in the material analyzed, whereas, in the actual material, the elemental composition may not be uniform.

### 3.3 Techniques to Determine Crystal Structures

**Figure 5:**
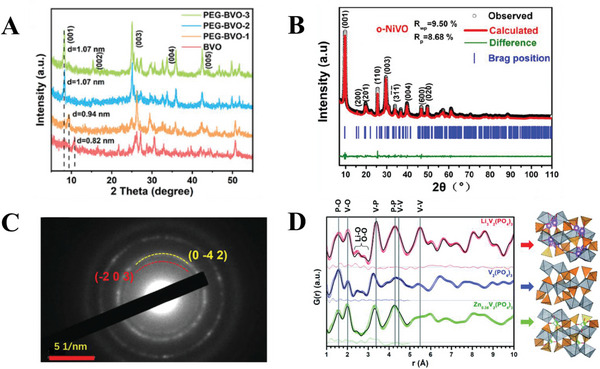
Examples of experimental techniques for the identification of structural changes during intercalation. A) XRD spectra for barium vanadium oxide (BVO) and BVO intercalated with polyethylene glycol (PEG‐BVO). Reproduced with permission.^[^ ^27^ ^]^ Copyright 2022, American Chemical Society. B) Rietveld refinement of the XRD spectrum of nickel‐intercalated vanadium oxide. Reproduced with permission.^[^ ^10e^ ^]^ Copyright 2022, Elsevier. C) SAED pattern of potassium and zinc intercalated vanadium oxide cathode. Reproduced with permission.^[^ ^21g^ ^]^ Copyright 2022, Wiley‐VCH. D) Pair distribution functions of vanadium phosphate materials intercalated with Li^+^ and Zn^2+^, calculated from WAXS data. Reproduced with permission.^[^ ^32^ ^]^ Copyright 2019, Royal Society of Chemistry.

Yin et al. developed a polyaniline‐intercalated vanadium oxide cathode, observing a shift of the XRD peak corresponding to the (001) plane of vanadium oxide to lower 2θ values upon the intercalation of polyaniline, representing an increase in the interlayer spacing caused by the inclusion of large polyaniline molecules.^[^ ^7a^ ^]^ Tan et al. observed a shift in the XRD peak of their VS_2_ cathode to lower 2*θ* values upon discharge, representing an increase in the interlayer spacing upon Zn^2+^ intercalation,^[^ ^7d^ ^]^ with Islam et al. observing a similar behaviour in their polypyrrole‐coated sodium vanadium oxide cathode.^[^ ^7f^ ^]^ Many works have similarly utilized XRD to observe expansion^[^ ^7^ ^]^ or contraction^[^ ^9^, ^10^ ^]^ of the lattice spacing of the cathode during Zn^2+^ intercalation in the discharge process. By looking at which of the peaks in the XRD spectra shift after intercalation, information about where the intercalated ions are inserted can be deduced. This has been applied to both Zn^2+^ intercalation,^[^ ^7^, ^10^, ^11^, ^20^, ^22^ ^]^ and other intercalated species,^[^ ^7^, ^10^, ^11^, ^14^, ^15^, ^21^, ^22^, ^27^ ^]^ with Jiang et al. observing a shift in the XRD spectra to lower angles upon the intercalation of polyethylene glycol into their barium vanadium pentoxide cathode (**Figure** **5**).^[^ ^27^ ^]^ XRD is a technique that operates based on the diffraction of X‐rays when incident and reflected off a crystal lattice. X‐rays reflected off different planes in the crystal structure have different phase shifts, and the superposition of these reflected rays gives rise to a diffraction spectrum. The angular position of each peak in the spectrum can be related to a parameter in the lattice spacings of the material via Bragg's equation. A shift in an XRD peak to higher values of 2*θ* indicates a contraction of the respective lattice spacing, and a downward shift in the 2*θ* value indicates an expansion. XRD spectra are also often compared to spectra in databases constructed from both experimental and simulation results to unambiguously confirm the identity of the cathode material,^[^ ^7^, ^9^, ^10^, ^11^, ^12^, ^16^, ^21^, ^22^, ^27^, ^28^ ^]^ or to identify new phases produced during the discharge process.^[^ ^7^, ^11^, ^12^, ^14^, ^15^, ^16^, ^21^, ^29^ ^]^ Additionally, Liu et al. conducted XRD on a thermally annealed Ni‐containing vanadium oxide electrode and used Rietveld refinement to fit the pattern to a model of Ni_0.6_V_1.9478_O_5_, with the fit of the theoretical model confirming the absence of water in the structure after the annealing process (Figure 5),^[^ ^10e^ ^]^ demonstrating how Rietveld refinement, a mathematical technique that fits a theoretical XRD spectrum to experimental results, has been used to enhance the quantitative power of XRD results.^[^ ^7^, ^10^, ^11^, ^13^, ^21^, ^22^, ^30^ ^]^ XRD can constitute direct evidence for the intercalation and deintercalation of species in a material, since it reflects the crystal structure and lattice spacing of the material, which may be altered during intercalation, such as through the formation of different phases or the expansion or contraction of interlayer spacings. One potential problem that could arise with XRD is incorrect sample preparation leading to inaccurate results; if the mounted sample is not sufficiently flat, the skewed sample surface will affect the angle at which the incident X‐ray beam is reflected, creating a shift in the peaks observed in the XRD spectrum.^[^ ^31^ ^]^ This shift could lead to an incorrect conclusion that lattice parameters have changed when it is not the case.

In addition to XRD, Tao et al. utilized selected area electron diffraction (SAED) to understand the crystal structure of their K_2_Zn_2_V_10_O_28_ cathode, obtaining d‐spacing values of 0.2278 and 0.268 nm for the (0–42) and (−203) planes (Figure 5).^[^ ^21g^ ^]^ Multiple studies have utilized SAED as a technique to probe the structure of zinc ion battery cathodes after the intercalation of guest species.^[^ ^7^, ^10^, ^11^, ^12^, ^15^, ^21^, ^33^ ^]^ SAED is a technique used in tandem with transmission electron microscopy, where the diffraction of the electron beam by the crystal lattice of the sample produces a diffraction pattern, which can be analyzed to determine the crystal structure and lattice parameters. For powder samples, the existence of many individual crystals^[^ ^34^ ^]^ in different orientations will lead to a ring‐like appearance of the diffraction pattern. However, electron diffraction has a greater uncertainty compared to X‐ray diffraction,^[^ ^34^ ^]^ hence lattice parameters can be more precisely identified through XRD which can be used to support SAED analysis. While XRD and SAED are generally more frequently used to understand the crystal structure of cathodes, other techniques such as wide‐angle X‐ray scattering (WAXS)^[^ ^21^, ^32^ ^]^ have been applied to understand intercalation in zinc ion battery cathodes as well. Park et al. utilized WAXS to supplement the XRD data for their vanadium phosphate cathodes intercalated with Li^+^ and Zn^2+^, with the WAXS results revealing the local structure of the material (in the range of 1 Å) after ion intercalation (Figure 5).^[^ ^32^ ^]^

### 3.4 Techniques to Determine Chemical Environments

The chemical environment of a species corresponds to a broad range of factors that affect the chemical behaviour of a species, such as the presence of covalent bonds, ionic interactions, non‐covalent interactions such as dispersion forces and ion–dipole interactions, solvent environment, solution pH, et cetera. For the purpose of identifying intercalation into zinc ion battery cathodes, of special interest would be the formation of bonds and interactions between the cathode and the intercalants, as well as the weakening or strengthening of bonds in the cathode caused by intercalation of the guest species, which in turn have implications on battery performance. For example, the formation of strong chemical interactions between intercalated Zn^2+^ and the cathode during discharge has been suggested to be unfavourable, as it reduces the reversibility of the intercalation process due to increased difficulty of deintercalation, which can also cause degradation of the cathode over multiple charge–discharge cycles.^[^ ^5a^ ^]^

**Figure 6:**
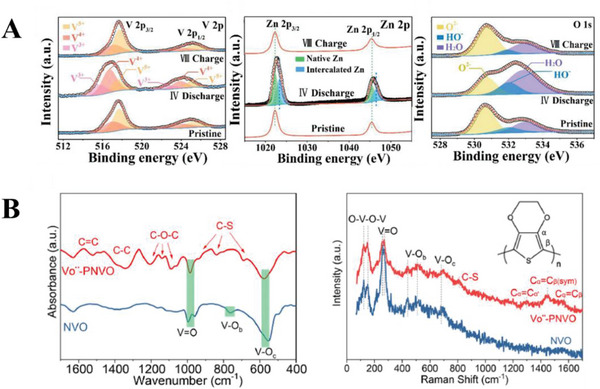
Examples of spectroscopic techniques used to characterize changes in the chemical environment of the cathode after intercalation. A) XPS spectra of zinc vanadium oxide, at various states. Reproduced with permission.^[^ ^9^ ^]^ Copyright 2022, Elsevier. B) FTIR (left) and Raman (right) spectra of sodium vanadate/PEDOT. Reproduced with permission.^[^ ^16^ ^]^ Copyright 2021, Elsevier.

Tong et al. detected the presence of Fe and N in the XPS spectrum of their vanadium oxide cathode, thus confirming the intercalation of iron and alkylammonium cations into their cathode material. They also concluded from XPS that the intercalation did not involve the formation of bonds with V atoms in the cathode, due to a lack of significant shifts in the vanadium XPS peaks.^[^ ^21f^ ^]^ Zhang et al. utilized XPS to identify the presence of Zn^2+^ during discharge, and a conversion of V from +5 to +4 oxidation states indicating reduction. They deconvoluted the O 1s spectra into components corresponding to the intercalation of water when solvated Zn^2+^ is inserted, and the formation of sulfur‐containing zinc hydroxide salts which indicated H^+^ intercalation (**Figure** **6**).^[^ ^9^ ^]^ In this way, XPS has been utilized by many studies to directly identify the presence of the intercalant by detection of the presence of an element found in the intercalant but not the pristine cathode.^[^ ^7^, ^10^, ^11^, ^15^, ^16^, ^20^, ^21^, ^29^ ^]^ XPS focuses an X‐ray beam on a sample, leading to the emission of photoelectrons via the photoelectric effect, with the energy of the photoelectrons being indicative of the binding energy of the atoms of the sample. The binding energy of the photoelectrons emitted not only indicates the presence of a certain element but also reflects the chemical state of that atom. Deconvolution of XPS peaks is often necessary to elucidate the individual signals separately when their peaks overlap, so as to obtain greater information about the cathode after intercalation and the intercalants. This process is based on the mathematics of the convolution theorem, where the convolution of two functions can be expressed as the product of their respective Fourier transforms.^[^ ^35^ ^]^ XPS has also been frequently used to observe intercalation indirectly by detecting changes in the chemical state of atoms in the cathode caused by intercalation of species, usually a decrease in the oxidation state of the cathode with Zn^2+^ intercalation during discharge.^[^ ^7^, ^9^, ^10^, ^11^, ^12^, ^21^, ^29^, ^36^ ^]^ Changes in the oxidation states of the cathode have also been observed by XPS to occur with the pre‐intercalation of aluminium ions,^[^ ^11e^ ^]^ ammonium ions,^[^ ^22^ ^]^ polyethylene glycol,^[^ ^27^ ^]^ polyaniline,^[^ ^15^ ^]^ and PEDOT.^[^ ^21c^ ^]^ XPS also allows for the calculation of the relative abundance of each species identified from the integral of the peaks in the XPS spectrum and has been used to calculate the stoichiometric formula of the intercalated material.^[^ ^10f^ ^]^ However, one limitation of XPS is that it is a surface technique and can only characterize elemental composition on the surface of the material.

Together with XPS, Ma et al. utilized Fourier transform infrared spectroscopy (FTIR) and Raman spectroscopy to identify changes in an ethylenediamine‐vanadium oxide cathode during discharge. The FTIR spectra revealed the weakening of peaks corresponding to C─H and C─N bonds, which was attributed to the chelation of Zn^2+^ by ethylenediamine. Raman spectroscopy displayed a new peak at 371.11 cm^−1^, corresponding to the Zn─N bond, confirming the presence of Zn^2+^ intercalated during discharge and the formation of coordination bonds between ethylenediamine and Zn^2+^.^[^ ^11d^ ^]^ Feng et al. synthesized a polypyrrole‐intercalated vanadium oxide cathode, detecting C─C and C─N bond vibrations in the FTIR and Raman spectra, thus confirming the successful intercalation of polypyrrole.^[^ ^21d^ ^]^ Similarly, Bi et al. observed C─C vibrations in the FTIR and Raman spectra of their sodium vanadate/PEDOT cathode, indicating the intercalation of PEDOT into their material (Figure 6).^[^ ^16^ ^]^ FTIR and Raman spectroscopy has often been used in tandem,^[^ ^9^, ^10^, ^11^, ^16^, ^21^ ^]^ to elucidate the chemical bonds and interactions present in a sample. FTIR spectroscopy involves the irradiation of the sample with infrared radiation, with the photons being absorbed by the bonds in the sample which are excited to higher energy rotational or vibrational states. The different rotation or vibration modes of different bonds or groups absorb photons of different characteristic energies, hence the FTIR absorption spectrum of a sample allows for the identification of the bonds or groups present in the sample. Raman spectroscopy involves irradiating a sample with electromagnetic radiation, with the incident photons interacting with the electronic profile of the sample, causing a shift in the energy of the photons. The extent of this shift depends on the polarizability of the electrons in the sample; hence, different shifts correspond to different chemical environments in the sample. However, the fitting and assignment of FTIR and Raman peaks are often slightly subjective, as larger systems lead to more complex spectra; for instance, the presence of intercalated water in various states of attraction to the material could lead to a broad FTIR peak from 3700 to 3500 cm^−1[^ ^37^ ^]^ which could obscure other signals in that region.

### 3.5 Other Experimental Techniques

**Figure 7:**
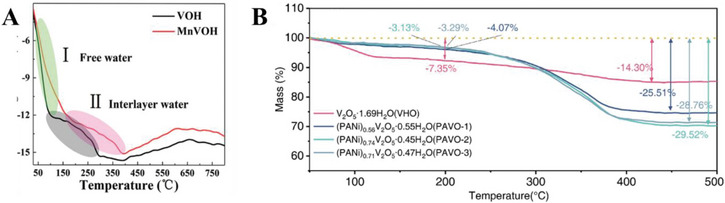
Examples of the use of TGA to characterize intercalated species in the cathode. A) TGA of unmodified and Mn‐intercalated hydrated vanadium oxide. Reproduced with permission.^[^ ^11a^ ^]^ Copyright 2021, Wiley‐VCH. B) TGA of hydrated V_2_O_5_ unmodified and pre‐intercalated with polyaniline. Reproduced with permission.^[^ ^11c^ ^]^ Copyright 2021, Elsevier.

Yin et al. conducted thermogravimetric analysis (TGA) on their polyaniline‐intercalated vanadium pentoxide cathode and observed a weight loss of 3.3% below 350 °C corresponding to the loss of adsorbed water, and a further loss of 5.4% corresponding to the loss of structural water. The weight loss of 7.8% at 400 °C was attributed to the thermal decomposition and evaporation of polyaniline.^[^ ^7a^ ^]^ Similarly, Li et al. synthesized a V_2_O_5_ cathode intercalated with polyaniline and observed two weight loss regimes corresponding to the loss of water and the decomposition of polyaniline (**Figure** **7**).^[^ ^11c^ ^]^ Chen et al. reported two stages in the TGA analysis of their hydrated manganese vanadium oxide cathode, corresponding to the loss of free water and the loss of interlayer water (Figure 7).^[^ ^11a^ ^]^ TGA involves measuring the mass of a sample as the temperature is increased, where a decrease in the mass of the sample while it is being heated corresponds to the loss of small molecules such as water, or the decomposition of the material. In the context of cathodes for aqueous zinc ion batteries, the initial weight loss at lower temperatures corresponds to the loss of water molecules. Further weight loss at high temperatures often indicates the decomposition of certain species, such as organic molecules pre‐intercalated into the cathode. The weight loss caused by the loss of water can be further divided into an initial decrease caused by the evaporation of adsorbed water, and a further decrease caused by the loss of structural water which is more strongly bonded to the material. TGA has been used quantitatively to estimate the content of water,^[^ ^10^, ^12^, ^21^ ^]^ or other molecular and carbon‐based intercalants in a cathode material.^[^ ^7^, ^11^, ^14^, ^16^, ^21^ ^]^ Nonetheless, due to the small mass decreases in many other cathode materials (often ⪅10% each for absorbed water and structural water^[^ ^7^, ^10^, ^11^, ^12^, ^21^, ^22^, ^27^ ^]^), TGA should not be considered as an effective quantitative technique for precise determination.

### 3.6 Electrochemical Techniques

While electrochemical methods may not directly reveal the presence of intercalants in a cathode material, changes in the electrochemical response of the cathode are indicative of structural and chemical changes in the material caused by the inclusion of intercalants. The electrochemical behaviour of the cathode has direct implications on the performance of the battery cell, making electrochemical techniques an indispensable characterization tool.

Feng et al. conducted cyclic voltammetry (CV) on their cell with a vanadium oxide/polypyrrole cathode, reporting two significant peaks at 0.73/0.57 and 1.01/0.97 V which were attributed to V^5+^/V^4+^ and V^4+^/V^3+^ respectively based on known values for these reactions. They observed the presence of smaller peaks at 0.73, 1.17, and 1.28 V which suggested a complex multi‐step intercalation and deintercalation of Zn^2+^ into the cathode. They also observed that their vanadium oxide/polypyrrole cathode exhibited smaller voltage differences between the pairs of cathodic and anodic peaks compared to unmodified vanadium oxide, indicating that the inclusion of polypyrrole into the cathode improved the kinetics of zinc intercalation.^[^ ^21e^ ^]^ Recently, Wu et al. reported the synthesis of a zinc vanadium oxide cathode with nitrogen and carbon motifs from the thermal annealing of metal–organic frameworks, observing in the cyclic voltammetry graph two pairs of peaks at 0.50/0.82 and 0.90/1.29 V, representing the intercalation of Zn^2+^ and H^+^.^[^ ^38^ ^]^ Chen et al. observed two pairs of redox peaks in the discharge of their V_3_O_7_ cathode, suggesting a two‐step mechanism for the intercalation of Zn^2+^ which was supported by computational simulations.^[^ ^39^ ^]^ Therefore, it can be noted that CV has been able to provide indirect information about the intercalation of species into the cathode during the discharge and charging processes. CV involves increasing and decreasing the applied potential across an electrochemical system in the range of a potential window and measuring the current, with current peaks indicative of the occurrence of pseudocapacitive redox reactions. Current peaks during the positive scan are indicative of oxidation processes occurring, and current peaks during the negative scan are indicative of reduction processes. Comparing the position and intensity of these peaks with the CV profiles of similar systems with well‐established reaction mechanisms can provide indirect evidence for one to propose an intercalation mechanism occurring in their cathode. Additionally, the kinetics of the pseudocapacitive processes in the cell can be deduced by the voltage difference in the anodic and cathodic peaks, with a smaller difference indicating greater reversibility and faster kinetics.^[^ ^21^, ^40^ ^]^

In addition, it has been proposed^[^ ^41^ ^]^ that varying the potential scan rate during CV provides information about the mechanism of charge storage in the cathode. The peak current *i* has been proposed to depend on scan rate *v* according to the equation

(1) $$i=av^{b}$$

where *a* and *b* are constants. A value of 0.5 for *b* suggests that charge storage is dominated by ion diffusion, whereas a value of 1 suggests the dominance of pseudocapacitive processes.^[^ ^42^ ^]^ Li et al. reported a *b* value of 0.79 to 0.87 for their MoS_2_ electrode inserted with N‐doped carbon motifs, which was higher than that of commercial MoS_2_, which was taken to indicate greater pseudocapacitive behaviour. This was attributed to the increased interlayer spacing of their material after the insertion of N‐doped carbon, which improved the kinetics and increased the surface area of the cathode for the pseudocapacitive intercalation of Zn^2+^ ions.^[^ ^11b^ ^]^ Zheng et al. reported that intercalation of Al^3+^ into a hydrated vanadium oxide increased the *b* value from 0.90 to 0.95, which could correspond to improved kinetics of the redox processes due to faster ion diffusion.^[^ ^40^ ^]^ Sun et al. and Chen et al. have similarly linked increased pseudocapacitive behaviour with improved Zn^2+^ intercalation kinetics and increased surface area for Zn^2+^ intercalation,^[^ ^43^ ^]^ since Zn^2+^ intercalation into the cathode is a faradaic process which is pseudocapacitive in nature.

**Figure 8:**
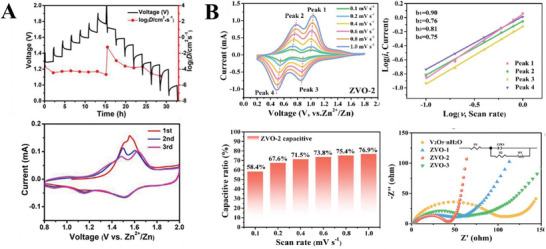
Examples of electrochemical techniques utilized to understand intercalation behaviour in the cathode. A) GITT profiles for a KVOPO_4_ cathode and the calculated diffusion coefficient, and cyclic voltammetry results at a scan rate of 0.1 mV s^−1^. Reproduced with permission.^[^ ^28^ ^]^ Copyright 2021, Elsevier. B) Cyclic voltammetry, fitted *b* values, calculated capacitive contributions, and Nyquist plots for zinc vanadium oxide cathode. Reproduced with permission.^[^ ^9^ ^]^ Copyright 2022, Elsevier.

In addition to CV, Zhu et al. utilized the galvanostatic intermittent titration technique (GITT), reporting that intercalation of K^+^ into their VOPO_4_ cathode increased the diffusion coefficient of Zn^2+^ by several orders of magnitude, attributing it to changes in the interlayer spacing after K^+^ intercalation (**Figure** **8**).^[^ ^28^ ^]^ Zhou et al. similarly conducted GITT on a potassium zinc vanadate cathode, observing a high diffusion rate greater than that for MnO_2_,^[^ ^21g^ ^]^ likewise Zhang et al. utilized GITT to conclude that vanadium oxide pre‐intercalated with tetramethylammonium ions had a higher Zn^2+^ diffusion coefficient compared to other V‐based cathodes.^[^ ^44^ ^]^ Multiple studies have utilized GITT to observe changes in the ion conductivity of zinc ion battery cathodes before and after the intercalation of guest species.^[^ ^7^, ^11^, ^21^, ^28^, ^33^, ^44^ ^]^ GITT involves the charge and discharge of the cell through intermittent current pulses. Based on the change in the cell voltage during the current pulses, the diffusion coefficient of the ions in the cell can be calculated.

Together with GITT, Yang et al. applied electrochemical impedance spectroscopy (EIS) to determine the conductivity of their cathode, observing that the charge transfer resistance of their potassium intercalated MnO_2_ cathode was lower when discharged to 1.4 V and higher when discharged to 1.24 V, which they attributed to H^+^ intercalation at 1.4 V which had faster kinetics of diffusion due to the smaller size of H^+^, compared to Zn^2+^ intercalation at 1.24 V.^[^ ^26^ ^]^ Jiang et al. also utilized EIS to demonstrate that the intercalation of polyethylene glycol resulted in a lower resistance of a barium vanadium oxide cathode,^[^ ^27^ ^]^ and similarly, Li et al. used EIS to determine that the resistance of a MoS_2_ cathode intercalated with nitrogen and carbon motifs exhibited a much lower resistance (60.08 Ω) than commercial MoS_2_ (257.5 Ω).^[^ ^11b^ ^]^ EIS has been used to understand the effects of intercalation on the ionic conductivity of the cathode,^[^ ^7^, ^11^, ^21^, ^26^, ^27^, ^28^, ^36^, ^40^, ^43^ ^]^ and is able to quantify ion conductivity by measuring the real (Z′) and complex (Z″) components of impedance at various frequencies of alternating current. The Nyquist plot of Z″ against Z′ for zinc ion battery cathodes usually features a sloping line at low frequencies representing ion diffusion resistance, a semicircle at medium frequencies representing charge transfer resistance, and another semicircle at high frequencies representing surface film resistance.^[^ ^27^ ^]^ Wu et al. utilized EIS in a novel method to calculate the kinetic barrier for Zn^2+^ ion intercalation. They quantitatively measured the charge transfer resistance of their hydrated ZnMn_2_O_4_ cathode at various temperatures using EIS, observing that the plot of charge transfer resistance against temperature obeyed the Arrhenius equation, following which the activation energy of Zn^2+^ ion insertion into the cathode was calculated.^[^ ^45^ ^]^ A combinatory approach of cyclic voltammetry, calculation of capacitive contributions by fitting the CV peak intensities and EIS has been utilized by Zhang et al. to understand the intercalation processes occurring in their zinc vanadium oxide cathode (Figure 8).^[^ ^9^ ^]^

### 3.7 Ab Initio Simulations

DFT has been by far the most common computational method used to simulate the behaviour of zinc ion battery cathodes. DFT is an ab initio (“from first principles”) simulation method based on the Kohn–Sham equation and is notably more computationally efficient than wavefunction‐based methods which predated it, making it suitable for the calculation of more complex solid‐state systems.^[^ ^46^ ^]^ Energetically stable structures of a chemical system can be obtained using DFT, by optimizing the distances between the atomic coordinates to achieve a minimum potential energy. Thus, DFT provides a theoretical method to calculate the precise structure of a material with given empirical information, such as its stoichiometric formula and crystal structure (obtained, for instance, from ICP‐AES^[^ ^10f^ ^]^ and XRD^[^ ^9^ ^]^). The most direct interpretation of DFT models would be observing the interatomic distances in the optimized unit cell before and after the inclusion of the intercalant.^[^ ^21^, ^39^, ^47^ ^]^ Chen et al. calculated the unit cell dimensions with and without the presence of Zn^2+^ intercalant, with the increased unit cell dimensions with the presence of Zn^2+^ used to substantiate experimental observation of lattice spacing expansion after intercalation;^[^ ^39^ ^]^ similarly, Liu et al. conducted DFT simulations which confirmed an increase in interlayer spacing of >10 nm upon intercalation of water in their Co‐doped MoS_2_ cathode.^[^ ^21k^ ^]^

**Figure 9:**
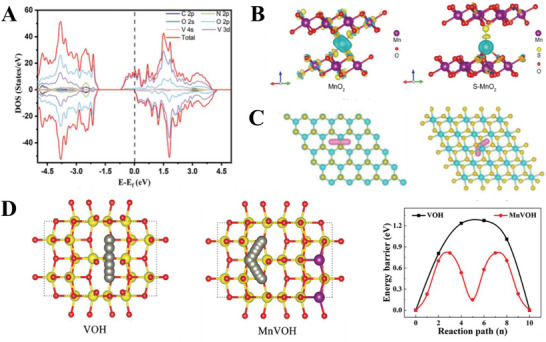
Examples of DFT are used to understand intercalation in the cathode. A) Density of states of ethylenediamine‐vanadium oxide simulated using DFT. Reproduced with permission.^[^ ^11d^ ^]^ Copyright 2022, Wiley‐VCH. B) Charge densities in MnO_2_ (left) and S‐doped MnO_2_ (right), calculated using DFT. Reproduced with permission.^[^ ^11g^ ^]^ Copyright 2022, Elsevier. C) Zn^2+^ diffusion pathway in 2H‐MoS_2_ and 1T‐MoS_2_ calculated using DFT. Reproduced with permission.^[^ ^21k^ ^]^ Copyright 2023, Elsevier. D) Zn^2+^ diffusion paths within unmodified and Mn‐doped hydrated vanadium oxide and the energy barriers for diffusion. Reproduced with permission.^[^ ^11a^ ^]^ Copyright 2021, Wiley‐VCH.

From a DFT model of their material, Ma et al. calculated the density of states of their ethylenediamine/vanadium oxide cathode, reporting that the inclusion of ethylenediamine causes the cathode to exhibit metallic conductivity with zero band gap. The resultant high conductivity explained the good performance of their material (**Figure** **9**).^[^ ^11d^ ^]^ Recently, Liu et al. reported the synthesis of a cobalt‐doped MoS_2_ cathode, utilizing DFT density of states calculations to demonstrate that the intercalation of Co caused a phase transformation from a semiconducting 2H‐phase to a 1T‐phase exhibiting metallic conductivity (Figure 9).^[^ ^21k^ ^]^ Using an optimized model of the material, density of states calculations have been used to understand the electronic properties of zinc ion battery cathodes.^[^ ^7^, ^10^, ^11^, ^21^, ^47^ ^]^ The density of states of a system indicates the permitted states that can be occupied by electrons at various energy levels and allows one to determine the Fermi level and band gap of a material, which correlates to the electronic behaviour of the material. In addition to the density of states calculations, Zhao et al. also calculated the charge distribution in their sulfur/MnO_2_ cathode compared to MnO_2_, reporting an increase in the electron density around the inserted Zn^2+^ ions in their S/MnO_2_ cathode, reducing the charge density of the Zn^2+^ ions. This reduced the electrostatic interaction between the Zn^2+^ ions and the cathode, therefore allowing for greater reversibility of charge storage (Figure 9).^[^ ^11g^ ^]^ Such computations of electron distribution in a cathode material after intercalation provide insight into the atomic charges, which affect the strength of the electrostatic interactions between the intercalants and other charged atoms of the cathode.

In addition, Ma et al. also utilized the nudged elastic band method to calculate the energy barrier for the diffusion of Zn^2+^ through their cathode, reporting a reduced energy barrier when ethylenediamine is present, which indicated more rapid intercalation of Zn^2+^ which improved the kinetic performance of the cathode.^[^ ^11d^ ^]^ Chen et al. similarly calculated the kinetics of Zn^2+^ diffusion through hydrated vanadium oxide with and without the presence of pre‐intercalated Mn^2+^, reporting a lower energy barrier for Zn^2+^ diffusion after manganese intercalation (Figure 9).^[^ ^11a^ ^]^ The nudged elastic band method has been used to simulate Zn^2+^ diffusion in cathode materials,^[^ ^7^, ^11^, ^39^ ^]^ and involves the tracing of diffusion paths of Zn^2+^ through a model of the cathode by optimizing the intermediate states along the diffusion pathway. The energy profile of the resultant path can be used to model the energy barrier for Zn^2+^ diffusion along that pathway. While computational simulations are not methods that directly identify or characterize the presence of intercalants, they are nonetheless useful in tandem with other experimental techniques like XRD and electrochemical techniques in understanding the mechanisms of ion diffusion, intercalation, and deintercalation in a material, as well as modelling the electronic properties of the material before and after intercalation. One thing to note is the importance of linking the theoretical results of DFT calculations to empirical results. With a complex system, there are likely many local minima for the potential energy of the system, translating to the existence of many stable structures. Hence, it is necessary to first elucidate the structure of the material through empirical means before the construction of the DFT model. It would be even more ideal to compare the optimised DFT model with parameters obtained through empirical means, such as HRTEM,^[^ ^11b^ ^]^ to verify the model before further calculations are performed.

## 4 Identifying Common Intercalants in Zinc Ion Battery Cathodes

In the previous section, we have identified the various characterization techniques that could be utilized in determining the presence of intercalants in the cathode material. In this section, we will discuss how these techniques were used in previous works in determining particular intercalant species. Including both intercalation processes during discharge and pre‐intercalation of species for cathode modification, intercalants into the cathode material of aqueous zinc ion batteries can be generally grouped into 4 categories: 1) metallic ions, 2) small molecules and non‐metallic ions, 3) polymers, and 4) the H^+^ ion.

### 4.1 Metallic Ions

The most common metallic intercalant into the cathode is Zn^2+^ as it often features in the charge storage mechanism of the battery. As earlier mentioned, due to the size of solvated Zn^2+^, in some materials, an expansion in the interlayer spacing of the cathode^[^ ^7^ ^]^ is observed. In other cathode materials, intercalation of Zn^2+^ results in a detectable contraction^[^ ^9^, ^10^ ^]^ in the lattice spacing of the cathode material owing to interactions between the bivalent Zn^2+^ cation and anionic centres in the material, or alternatively explained by the replacement of intercalated water molecules by smaller Zn^2+^ ions.^[^ ^10f^ ^]^ These changes in the interlayer spacing have often been observed through XRD where a shift in an XRD peak to a smaller 2θ after Zn^2+^ intercalation corresponds to an expansion in the interlayer spacing, and vice versa. By observing which peak had shifted, the plane where Zn^2+^ intercalates could be deduced.^[^ ^7^, ^9^, ^10^, ^11^, ^14^, ^16^, ^20^, ^21^ ^]^ In addition, phase changes in the cathode material due to the intercalation of Zn^2+^ could occur with the formation of a Zn‐containing phase. The emergence of such new phases has been observed through the formation of new peaks in the XRD spectrum and a decrease in the intensities of peaks corresponding to the original structure.^[^ ^7^, ^9^, ^10^, ^11^, ^15^, ^21^ ^]^

Additionally, the intercalation of Zn^2+^ ions during discharge has often been accompanied by a reduction in the oxidation states of the cathode, which is often observed using XPS,^[^ ^7^, ^9^, ^10^, ^11^, ^12^, ^21^, ^29^ ^]^ and the presence of Zn has been directly observed from the presence of its characteristic signals in the XPS spectrum.^[^ ^7^, ^9^, ^10^, ^22^ ^]^ Furthermore, if strong bonds are formed between intercalated Zn^2+^ and the anionic centres of the cathode, signals corresponding to these bonds can be identified in the Raman^[^ ^9^, ^11^, ^29^, ^44^ ^]^ or XPS^[^ ^11d^ ^]^ spectra of the cathode after discharge; for instance, Zhao et al. reported the detection of peaks in the Raman spectra of their MnO_2_‐based cathode after discharge that corresponded to the formation of Zn─O bonds, confirming the intercalation of Zn^2+^ and suggesting a strong interaction between intercalated Zn^2+^ and their cathode.^[^ ^29a^ ^]^ Zn^2+^ intercalation could also modify the geometry and energy of existing chemical bonds in the cathode, which could be observed as shifts in the peaks, or changes in peak intensities, of FTIR,^[^ ^11^, ^21^, ^29^ ^]^ Raman^[^ ^7^, ^10^, ^11^, ^21^ ^]^ and XPS^[^ ^10^, ^11^ ^]^ spectra. For example, Ma et al. conducted FTIR on their ethylenediamine‐vanadium oxide cathode after full discharge and observed a weakening of the peaks corresponding to C─H, C─N and V─O bonds, and a blueshift of the peak corresponding to N─H. These changes were attributed to the chelation of intercalated Zn^2+^ by ethylenediamine.^[^ ^11d^ ^]^

Other metallic ions have also been pre‐intercalated into cathode materials to improve performance, with transition metals such as Cu,^[^ ^7b^ ^]^ Ni^[^ ^10^, ^21^ ^]^ and Mn;^[^ ^11a^ ^]^ alkali metals such as K^[^ ^7c^ ^]^ and Li;^[^ ^21b^ ^]^ and other metals such as Ca^[^ ^10^, ^27^ ^]^ and Al^[^ ^11e^ ^]^ having been used as intercalants. Chen et al. reported a novel metal–organic framework cathode material for an aqueous nickel–zinc batteries, and a synthesis strategy for the pre‐intercalation of metal cations such as Mn^2+^, Co^2+^, Cu^2+^, Zn^2+^, Al^3+^, and Fe^3+^; specifically, the intercalation of Co^2+^ achieved an improvement in the stability of the cathode over 10 000 charge/discharge cycles, and good specific capacity and energy density.^[^ ^48^ ^]^ The intercalation of metallic ions can improve the kinetics of Zn^2+^ intercalation during battery discharge and stabilize the cathode for better capacity retention over many cycles. DFT calculations by Chen et al. revealed an increase in the interlayer spacing when Mn^2+^ was intercalated into a vanadium oxide host material, which facilitated the intercalation of Zn^2+^ during discharge, leading to improved energy density. Additionally, calculations pointed to the formation of strong bonds between the inserted Mn^2+^ and the vanadium oxide host material, stabilizing the material and accounting for the high capacity retention over 5000 cycles.^[^ ^11a^ ^]^ The intercalation of metallic ions can also improve the conductivity of the cathode and the thermodynamic favourability of Zn^2+^ storage, improving the electrochemical performance of the cathode. In recent work, Lv et al. investigated a vanadium oxide cathode intercalated with Al^3+^ and utilized DFT calculations to demonstrate that Al^3+^ intercalation into the cathode material increased the density of states near the Fermi level, accounting for the improved conductivity observed in experimental results. The material intercalated with Al^3+^ also exhibited a more negative binding energy of Zn^2+^ in DFT calculations, suggesting greater thermodynamic favourability of Zn^2+^ storage.^[^ ^49^ ^]^

Similar to Zn^2+^ intercalation, changes in lattice spacing upon the pre‐intercalation of metal ions can be observed via XRD. The result could be a decrease in lattice spacing when small ions such as Al^3+^ are intercalated,^[^ ^11e^ ^]^ or an increase in lattice spacing for larger ions such as Ca^2+[^ ^27^ ^]^ and Fe^3+^.^[^ ^21f^ ^]^ Added intercalants can also result in a decrease in the crystallinity of the material due to the distortion in the lattice structure caused by the intercalants; this can be observed as a decrease in the intensity of the XRD peaks.^[^ ^7^, ^9^, ^10^ ^]^ Interactions formed between the intercalant ions and the cathode can also be observed as additional peaks in the Raman spectra in the case of the formation of new bonds,^[^ ^10^, ^40^, ^47^ ^]^ or shifts in the original Raman spectra representing changes in bond energies of the cathode after guest ion intercalation.^[^ ^10^, ^30^, ^40^ ^]^

### 4.2 Hydrogen Ions

The intercalation of H^+^ has been identified as a possible charge storage mechanism in aqueous zinc ion batteries.^[^ ^4^, ^5^ ^]^ If H^+^ is intercalated into the cathode, the OH^−^ remaining in the electrolyte will result in an increase in electrolyte pH, which could cause the precipitation of zinc hydroxide compounds as a by‐product. Hence, by observing if the characteristic diffraction peaks of zinc hydroxide salts appear during discharge, one is able to indirectly determine if H^+^ is intercalated into the cathode.^[^ ^7^, ^10^, ^16^, ^21^, ^29^ ^]^ During the discharge process of their vanadium oxide‐PEDOT cathode, Kim et al. observed through in situ XRD the formation of new diffraction peaks at 6°, 12°, and 33°. The cathode material was then removed, washed and then observed under XRD, and peaks corresponding to zinc hydroxide triflate were observed, signalling the intercalation of H^+^ into the cathode had occurred during discharge.^[^ ^10c^ ^]^ Not all zinc ion batteries exhibit H^+^ intercalation as a charge storage mechanism; Chen et al. reported the absence of the production of zinc hydroxide salts on their manganese vanadium oxide cathode during discharge, leading them to conclude that H^+^ was not intercalated into the cathode during discharge.^[^ ^11a^ ^]^ In addition, for MnO_2_ based cathodes, the detection of an MnOOH phase by XPS^[^ ^29a^ ^]^ or XRD^[^ ^29^, ^33^ ^]^ also provides evidence for the intercalation of H^+^.

### 4.3 Small Molecules and Non‐Metallic Ions

Synthesis of cathodes in aqueous conditions and the immersion of cathodes in the aqueous electrolyte often result in the inclusion of intercalated water molecules in the material,^[^ ^10^, ^11^, ^22^ ^]^ which could result in an expanded lattice spacing compared to the dehydrated material.^[^ ^13c,f^ ^]^ Cui et al. reported an increase in the interlayer spacing of their NH_4_V_4_O_10_ cathode as observed with in situ XRD when it was immersed in the aqueous electrolyte for 4 h.^[^ ^11f^ ^]^ There are often two types of intercalated water molecules present in the material that can be identified via TGA: water molecules physically adsorbed on the material, which is the first to be released in TGA; and water molecules more strongly bonded to the material, which is only released upon further heating.^[^ ^7^, ^10^, ^11^, ^21^, ^22^ ^]^ Additionally, the intercalation of Zn^2+^ during discharge has often been accompanied by the insertion of water molecules,^[^ ^7^, ^10^, ^11^, ^15^, ^21^ ^]^ due to the high valence of Zn^2+^ which causes it to exist in aqueous solutions with a solvation shell of water molecules. This co‐intercalation of water can be detected using FTIR^[^ ^21k^ ^]^ and XPS;^[^ ^7^, ^10^, ^11^, ^21^, ^22^, ^45^ ^]^ Li et al. reported that the XPS O1s peak assigned to H_2_O in their polyaniline ammonium vanadate cathode increased in intensity concurrently with the insertion of Zn^2+^ during discharge, and weakened during charging, indicating the co‐insertion (and removal) of H_2_O with Zn^2+^.^[^ ^15^ ^]^ The insertion of water molecules with Zn^2+^ is considered favourable as it could reduce the energy barrier for diffusion of Zn^2+^ in the cathode as evidenced by DFT calculations.^[^ ^11a^ ^]^ This is because the presence of water molecules shields the positively charged Zn^2+^ ion from anionic centres in the cathode, as well as the expanded interlayer spacing caused by the intercalated water molecules.^[^ ^11c^ ^]^

The pre‐intercalation of small molecules and polyatomic ions has also been applied as a strategy to modify the cathode material in order to improve capacity and cycling performance. Recent examples include carbon dioxide,^[^ ^50^ ^]^ ammonia,^[^ ^10a^ ^]^ ammonium ions,^[^ ^15^, ^43^ ^]^ ethylenediamine,^[^ ^11d^ ^]^ and *N, N*‐dimethylformamide.^[^ ^14^ ^]^ The intercalation of small molecules usually serves to expand the interlayer spacing of the cathode and also acts to shield charged centres to reduce the strength of the interactions between Zn^2+^ and the cathode, achieving improved kinetics of Zn^2+^ diffusion. For instance, Wan et al. reported an increase in the interlayer spacing of aluminium vanadate from 9 to 11.8 Å upon the intercalation of *N, N*‐dimethylformamide.^[^ ^14^ ^]^ Similarly, Ma et al. reported that the intercalation of ethylenediamine in a vanadium oxide cathode resulted in higher calculated adsorption energy for Zn^2+^,^[^ ^11d^ ^]^ indicating that the intercalation of ethylenediamine weakened the interaction between Zn^2+^ and the cathode, leading to improved Zn^2+^ diffusion kinetics.^[^ ^11d^ ^]^ Other non‐metallic ions have also been explored as intercalants, for instance, Zhao et al. reported the intercalation of S^2−^ in their MnO_2_ cathode.^[^ ^11g^ ^]^ Due to the covalent bonds in small molecules and polyatomic ions that will show up in the FTIR spectra of the material, FTIR^[^ ^11^, ^21^, ^44^, ^50^ ^]^ can be used to identify the presence of such intercalants. For example, Ma et al. conducted FTIR on their ethylenediamine‐vanadium oxide cathode, observing characteristic peaks at 1610, 1490, and 1330 cm^−1^ that were assigned to the vibrational modes of N─H, C─H and C─N bonds in ethylenediamine.^[^ ^11d^ ^]^

### 4.4 Polymers

Intercalation of organic polymers into zinc ion battery cathodes has also been explored, with studies focusing on conductive polymers such as polyaniline,^[^ ^7^, ^11^, ^33^, ^43^ ^]^ polypyrrole,^[^ ^21d,e^ ^]^ PODA (poly(4,4‐oxybisbenzenamine)),^[^ ^29a^ ^]^ and PEDOT (poly(3,4‐ethylenedioxythiophene)),^[^ ^10^, ^16^, ^21^ ^]^ due to their ability to improve the electrical conductivity of the cathode. The larger sizes of these polymers could also expand the interlayer spacing of the cathode, as well as stabilize the cathode through the formation of interactions between the cathode and the functional groups of the polymer, thus achieving improved Zn^2+^ diffusion kinetics and cycling stability respectively. Zhao et al. reported the synthesis of a MnO_2_ cathode inserted with PODA, with DFT calculations suggesting that the C═N bonds in PODA were able to coordinate to Zn^2+^ and stabilize the cathode during charge–discharge cycles. Additionally, the presence of PODA increased the energy barrier for the removal of Mn atoms from the cathode, suggesting that PODA could also suppress cathode dissolution.^[^ ^29a^ ^]^

Due to the presence of covalent bonds in polymers, FTIR^[^ ^7^, ^10^, ^15^, ^16^, ^21^, ^33^, ^43^ ^]^ and Raman^[^ ^16^, ^21^, ^27^, ^33^ ^]^ spectroscopy has been used to identify the intercalated polymer into the cathode. For instance, Kumankuma–Sarpong et al. reported the synthesis of a yttrium vanadium oxide cathode with inserted PEDOT, which presence was confirmed by the characteristic peak for C═C vibration in the Raman spectra, and peaks assigned to C─C, C═C and C─S observed in the FTIR spectra.^[^ ^21c^ ^]^ Observation of characteristic peaks in the XPS spectra corresponding to certain organic functional groups can also act as evidence for polymer intercalation;^[^ ^11^, ^43^ ^]^ for example, Li et al. observed peaks corresponding to the C─N and ─NH_2_ groups in their XPS spectrum of the cathode, concluding that successful intercalation of polyaniline had occurred.^[^ ^11c^ ^]^ Due to the formation of interactions and redox reactions between intercalated polymers and the cathode, the XPS peaks of the cathode can also change after intercalation;^[^ ^33^, ^43^ ^]^ for instance, Wang et al. reported that the intercalation of polyaniline in their vanadium oxide hydrate cathode increased the XPS signal corresponding to V^4+^, representing a partial reduction of V^5+^ in their cathode.^[^ ^33b^ ^]^

## 5 Perspectives and Outlook

Works investigating the intercalation of species into zinc ion battery cathodes often combine multiple characterization techniques to conclusively identify the presence of intercalants as well as deducing the mechanisms of intercalation. We believe that it is important to gain an understanding of the intercalation of species into newly synthesized zinc ion battery cathodes during discharge (and deintercalation during charging), including the mechanism of intercalation and the resultant changes in material structure and electronic properties. This is because the aim of designing new battery cathode materials should not be merely improved performance in isolation, but in creating an overall cell with improved battery performance, which requires an understanding of intercalation to decide the combination of cathode material and electrolyte under realistic operating conditions. It is in this regard that we believe understanding intercalation mechanisms can aid in directing research efforts in zinc ion battery cathode and electrolyte to improve overall battery performance. Any one technique is unable to conclusively prove intercalation if used in isolation; indirect methods such as observing changes in crystal structure with XRD or observation of CV profiles are unable to directly reveal the presence of intercalants, whereas direct identification of the intercalants with methods such as EDS is unable to reveal information such as the lattice plane in which the intercalants are inserted into, and the chemical interactions formed between the material and the intercalants. Computational methods are able to reveal the interactions that the cathode forms with the intercalants, as well as changes in the electronic structure of the material, but simulating large systems is computationally costly, and calculations based on smaller representative units have to be corroborated with experimental results. Additionally, intercalation often occurs in complex chemical environments; during battery operation, the intercalation into the cathode of multiple species found in the aqueous electrolyte such as Zn^2+^, H^+^ and H_2_O can simultaneously occur.^[^ ^4^, ^5^ ^]^ Therefore, we believe a combinatory approach is always necessary when studying intercalation, with multiple techniques utilized in tandem to reveal different aspects of the structure and chemistry of the material after intercalation. For instance, while SEM‐EDS is able to provide a visual map of the elemental composition of the surface of the material, XPS is able to provide information on the chemical environment of the elements on the surface of the material. The spatial distribution of the elements and information about their chemical states can then be combined to provide a convincing explanation of the mechanism of intercalation. Likewise, DFT calculations in isolation lack persuasiveness due to the complex nature of cathode materials, which leads to difficulty in determining if the optimized state achieved by a certain combination of basis set and functional truly reflects the structure of the material. Hence, DFT models require validation with experimental parameters and an agreement between DFT calculations and experimental results are required to present a sufficiently convincing explanation for cathode behaviour during intercalation.

To conclude with an overarching perspective on the strategies that can be employed to understand intercalation in battery cathodes, we believe that future works should supplement direct identification of the intercalant (through methods such as EDS or ICP‐AES) with XRD, as it provides a means of understanding any structural changes during intercalation, and with XPS, which is useful in determining changes in the chemical environment and oxidation states of the material. We believe that on top of these essential techniques, Raman and FTIR spectroscopy can also be utilized to further understand the chemical changes in the material, and DFT simulations can provide an explanation for the effects of intercalation based on fundamental chemical and physical interactions.

Finally, while in situ and ex situ characterization methods have been employed to study intercalation in zinc ion battery cathodes, and often a combination of both, there has been no critical discussion on the reliability of ex situ techniques compared to in situ techniques in providing evidence of intercalation. While ex situ techniques are often more convenient and do not require alterations to equipment for successful data collection, there remain concerns that ex situ sample preparation and potential contamination could alter the structural and chemical properties of the cathode,^[^ ^51^ ^]^ thus ex situ results may not be reflective of the material in operando. This is even more pertinent for zinc ion batteries utilizing aqueous electrolytes, since the intercalation of hydrated Zn^2+^ ions into the cathode often leads to the inclusion of water molecules in the cathode material.^[^ ^10^, ^11^, ^22^ ^]^ Additionally, the intercalation of hydrogen ions into the cathode often leads to the formation of hydrated zinc hydroxide salts; for instance, Jo et al. utilized in operando synchrotron XRD and Raman spectroscopy to verify the presence of a hydrated zinc hydroxyl triflate salt formed on the cathode of an aqueous zinc ion battery by intercalation and removal processes which occurred during battery operation.^[^ ^52^ ^]^ The evaporation of water content from these materials during sample preparation for ex situ analysis could affect the structure and chemical properties of the material, thus rendering ex situ analysis less accurate. In addition, in situ and specifically in operando methods would be useful to observe processes which occur gradually during battery operation such as changes in material crystallinity.^[^ ^53^ ^]^ In a study by Zhao et al., in operando X‐ray absorption spectroscopy was utilized to examine the changes in the chemical environment of an ammonium vanadium oxide cathode during the operation of an aqueous zinc ion battery cell. This allowed for the observation of changes in the geometry of the crystal structure and changes in the oxidation state of the vanadium centers during battery operation. In addition, in operando XRD was used to systematically elucidate the changes which occurred at the cathode during discharge in detail, identifying the occurrence of 2 solid‐solution reactions and 2 two‐phase transitions.^[^ ^54^ ^]^ Despite the abovementioned advantages of in situ methods, engineering challenges have to be overcome, such as incorporating X‐ray transparent windows for in situ XRD and the possible need for inert atmosphere conditions to prevent contamination when the material is exposed to the environment during in situ characterizations.^[^ ^53^ ^]^

## 6 Conclusion

In this review, we have discussed the intercalation and deintercalation of ions such as Zn^2+^ and H^+^ into the cathode as the basic charge storage mechanism of zinc ion batteries, and how intercalation of other guest species has been utilized to enhance cathode performance. We discussed the techniques utilized in existing works in literature to identify the presence and understand the intercalation mechanisms of these intercalants, dividing the techniques into 1) imaging techniques, 2) elemental identification techniques, 3) techniques to determine crystal structures, 4) techniques to determine chemical environments, 5) other experimental techniques, 6) electrochemical techniques, and 7) ab initio simulations. We then discussed the techniques that can be used to understand the intercalation of four general categories of intercalants: 1) metallic ions, 2) small molecules and non‐metallic ions, 3) polymers, and 4) the H^+^ ion, highlighting a few common approaches for each kind of intercalant, with the techniques used for each intercalant depending on their structure and the interactions formed during intercalation. We outlined our perspective on this topic by discussing the need for a combinatory approach in understanding intercalation. We suggest how future works can utilize a combination of techniques in understanding intercalation, outlining the importance of a rigorous understanding of intercalation to be able to holistically design zinc ion batteries with good overall performance.

## Conflict of Interest

The authors declare no conflict of interest.
